# Arm activity measure (ArmA): psychometric evaluation of the Swedish version

**DOI:** 10.1186/s41687-021-00310-4

**Published:** 2021-05-12

**Authors:** Therese Ramström, Lina Bunketorp-Käll, Johanna Wangdell

**Affiliations:** 1 Centre for Advanced Reconstruction of Extremities, Sahlgrenska University Hospital/Mölndal, House U1, Level 6, SE-431 80 Mölndal, Sweden; 2 Department of Hand Surgery, Institute of Clinical Sciences, Sahlgrenska Academy, University of Gothenburg, Gothenburg, Sweden; 3 Department of Health and Rehabilitation, Institute of Neuroscience and physiology, Sahlgrenska Academy, University of Gothenburg, Gothenburg, Sweden

**Keywords:** Central nervous system; spasticity; patient reported outcome measures, Spinal cord injury, Stroke traumatic brain injury, Rehabilitation, Upper limb

## Abstract

**Background:**

Patient Reported Outcomes Measure (PROM) are commonly used in research and essential to understand the patient experience when receiving treatment. Arm Activity Measure (ArmA) is a valid and reliable self-report questionnaire for assessing passive (section A) and active (section B) real-life arm function in patients with disabling spasticity. The original English version of ArmA has been psychometrically tested and translated into Thai.

**Aims:**

Translate and cross-culturally adapt ArmA to Swedish language and context. Further, to evaluate the reliability, validity and sensitivity of the Swedish version of the questionnaire (ArmA-S) in patients with disabling upper limb spasticity caused by injuries to the central nervous system (CNS).

**Materials and methods:**

ArmA was translated and cross-culturally adapted according to established guidelines. Validity and reliability were evaluated in 61 patients with disabling spasticity. Face and content validity was evaluated by expert opinions from clinicians and feedback from patients with upper limb spasticity. Internal consistency reliability was assessed with Cronbach’s alpha and test-retest reliability was assessed using the quadratic weighted kappa.

**Results:**

ArmA-S was shown to be clinically feasible, with good face and content validity and no floor or ceiling effects. Internal consistency of ArmA-S was high and equivalent to ArmA; with Chronbach´s alpha coefficients values of 0.94 and 0.93 for section A and B, respectively. Test-retest reliability was good, with kappa values of 0.86 and 0.83 for section A and B, respectively. Some layout modifications of ArmA-S were made to further increase the user-friendliness, test-retest reliability, and responsiveness.

**Conclusion:**

ArmA-S was shown to be a reliable and valid self-report questionnaire for use in clinical practice and research to assess improvements in passive and active upper limb function in patients with disabling spasticity.

**Supplementary Information:**

The online version contains supplementary material available at 10.1186/s41687-021-00310-4.

## Introduction

Spasticity after an injury to the central nervous system (CNS) can cause profound disability [1]. The prevalence of spasticity differs among various diagnoses, depending on how it is defined. Spasticity is reported to be present in 80% of patients with spinal cord injury (SCI) [2, 3], 60% of patients with traumatic brain injury (TBI) [4], and 30% of patients with stroke [5, 6]. The consequences of spasticity in the upper limb (UL) range from reduced grip control to a clenched fist and can prevent prehension and grasp, which are critical for independence in activities of daily living (ADL) [7]. Left untreated, spasticity can lead to severe contractures, deformity, pain, and involuntary movement and severely compromise occupational performance [3, 9–11]. Since being active is fundamentally important for all living beings, and participation in activities is necessary for human physical and mental wellbeing [8], disabling UL spasticity can have devastating consequences.

Patients with disabling spasticity are a heterogeneous group, and treatment goals differ depending on the degree of neurological impairment. For patients with residual volitional motor function, treatment often focuses on restoring active functions, whereas for those with more severe motor impairment, it focuses on improving passive everyday functional tasks, such as personal care (e.g., hygiene, dressing) Thus, to capture improvements resulting from various spasticity treatments, the outcome measure must include both passive and active aspects of everyday life.

Various measures can be used to assess the effects of spasticity on body function [9–12], but very few encompass both passive and active functional aspects. Even though these two functional constructs should be treated as separate entities, both are important in patient management. The lack of a comprehensive measure that is sensitive to change in patients with disabling UL spasticity resulted in the development of the Arm Activity Measure (ArmA) [13]. The original English version of ArmA has been carefully evaluated and is a valid, reliable and responsive self-report questionnaire for assessing real-life arm function after focal therapy intervention, and in particular spasticity interventions [14, 15]. ArmA can be done by the patient or a caregiver. ArmA has previously been translated into Thai [16]. For use in a Swedish context, ArmA must be translated and cross-culturally adapted to ensure that the Swedish version is semantically and conceptually equivalent to the original version. In this study, our goal was to translate ArmA into Swedish and adapt it to a Swedish context. We also evaluated the reliability by analysing internal consistency and test-retest of the measurement tool. Validity was assessed by face and content analyses, acceptability and construct validity, and responsiveness was assessed by longitudinal validity in a sample of Swedish-speaking patients with problematic UL spasticity after CNS injury.

## Materials and methods

### Translation and adaptation of the ArmA to a Swedish context

**Fig. 1:**
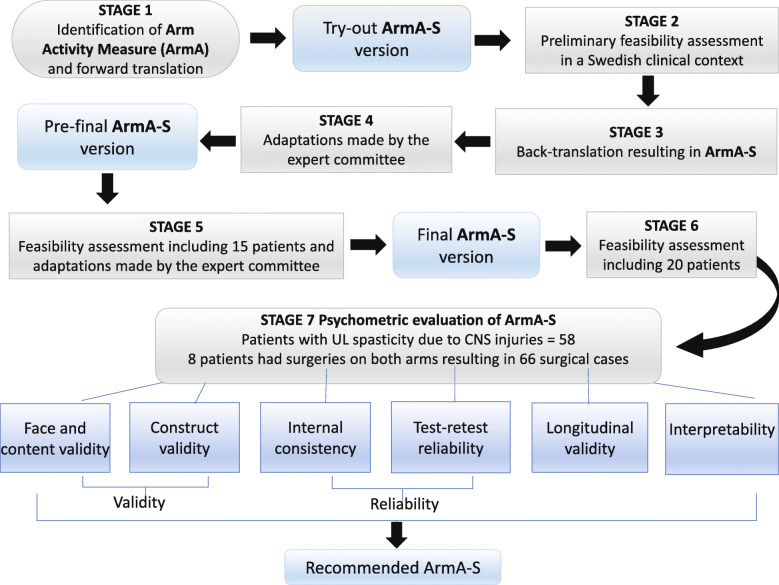
Description of study design

The developers of ArmA gave us permission (by correspondence with the first author in August 2017) to translate it into Swedish with a forward-back-translation procedure. To achieve equivalence between the original version of ArmA and the Swedish version (ArmA-S), ArmA was translated and cross-culturally adapted to the Swedish language and context, using the Beaton guidelines for translation of self-report health questionnaires [17]. The translation process is summarized in Fig. 1 and is described in detail in the Additional file 1.

#### Measures

##### The ArmA questionnaire

The ArmA is a self-report questionnaire used to measure the difficulty of passive and active UL daily tasks, referred to as passive and active function, in patients with unilateral paresis. The questionnaire was initially developed and psychometrically evaluated by the developer as a seven-item passive function subscale (section A) and a 13-item active function subscale (section B) [14, 16]. The section A was modified by the developer who added an item to be included in an eight-item version of the ArmA. This eight-item scale is recommended version and the one used in the present study (English version available for download on https://www.kcl.ac.uk/cicelysaunders/research/outcome/rehabilitation/arma). ArmA uses a five-point Likert scoring system, from 0 (*no difficulty*) to 4 (*unable to do the task*). The respondent is asked to circle the most appropriate response (0–4). Section B covers both unimanual and bimanual activities. Some of the unimanual activities are mostly done with the dominant hand. In the written instructions for the original version of ArmA, the respondent is asked to take the following into account when selecting a response option: If the task is *never done*, but this has nothing to do with your arm, please score difficulty as 0 (*no difficulty*). The passive function subscale (section A) scores range from 0 (high function) to 32, and the active function subscale (section B) scores range from 0 (high function) to 52. The subscales are analysed separately and may not be combined into a single sum score. The original version of ArmA is reliable and valid in patients with UL spasticity due to stroke and TBI and in those with other neurological injuries [15].

##### Other measures

To evaluate construct validity and responsiveness, we collected the following outcome measures at baseline and 3 months after spasticity-correcting surgery. UL spasticity was quantified with the modified Ashworth scale (MAS) [18]. For analysis, MAS scores were summed to provide a ‘composite spasticity score’. The grasp and release test (GRT) was used to assess the patient’s ability to manipulate objects typically used in ADL. In the GRT, the patient is asked to pick up, move, and release six objects of different sizes, weights, and textures using a palmar or lateral grasp [19]. The disability of the arm, shoulder, and hand (DASH) self-rated questionnaire (items 1–21) was used to assess the patient’s ability to perform activities during the previous week [20]. The first 21 items of DASH assess the difficulty of performing activities because of UL problems. Each activity item is scored 0 (*no difficulty*) to 5 (*extreme difficulty*). To achieve a score comparable to that of the ArmA questionnaire in the evaluation of construct validity, we used the same strategy as in the initial evaluation of ArmA, in which a total score was calculated for the summated active function items (items 1 to 21).

#### Participants

The study population consisted of patients with UL spasticity due to CNS injuries who were referred to Centre of Advanced Reconstruction of Extremities (CARE), Sahlgrenska University Hospital. The patients were consecutive recruited between September 2017 and April 2020. According to a treatment algorithm at CARE, patients were allocated to one of three treatment regimens—high-, low-, or non-functioning (HFR, LFR, NFR)—based on the patient’s remaining sensorimotor control in the UL and on cognitive ability. The treatment regimens are presented in previous studies [21, 22]. The inclusion criteria for the treatment regimens are presented in Table 1. The exclusion criteria were age < 18 years, inability to complete questionnaires because of language difficulties, or cognitive impairment and the absence of a caregiver or relative to complete the questionnaire.

**Table 1:** Description of inclusion and exclusion criteria for the different treatment regimens

Criteria	HFR	LFR	NFR
Inclusion criteria			
Muscle hypertonicity is the primary component of spasticity	x	x	x
The UL spasticity limits ADLs	x	x	x
The patient had nonpharmacologic and/or pharmacologic spasticity treatment, with specific recommendations for botulinum toxin injection	x	x	x
The patient has volition motor function in the UL	x	x	
The patient agrees to comply fully with the treatment regimen	x	(x)	
The patient is motivated to participate in intensive rehabilitation	x	(x)	
The patient has stable home care/assistance	x	(x)	
Functional score^a^ 1			x
Functional score^a^ 2		x	x
Functional score^a^ 3	x	x	(x)
Functional score^a^ 4	x	(x)	(x)
The patient must have residual shoulder mobility	x		
Exclusion criteria			
Severe cognitive impairments	x	x	
Mild cognitive impairments	x		
Severe contractures that hinder surgical benefit	x	(x)	

*HFR* High-functioning regimen, *LFR* Low-functioning regimen, *NFR* Non-functioning regimen

^a^= Mertens P, S.M., Surgical management of spasticity, in *Upper Motor Neuron Syndrome and Spasticity: Clinical Management and Neurophysiology*, J.G.E. Barnes MP, Editor. 2001, Cambridge University Press: Cambridge. pp. 239–65

### Data collection and test-retest reliability procedure

All authors participated in data collection. For the test-retest reliability procedure, the questionnaire was sent by mail to be completed 1 week before a scheduled visit to the clinic for the re-test. Alternatively, the questionnaire was sent by mail, and once returned, it was sent again 1 week later. No specific treatment was given between the two evaluations. Responsiveness was testing done at the clinic the day before the spasticity-correcting surgery, and again 3 months after surgery. The 3-month follow-up was done at the clinic or by mail.

### Data analyses

Demographic and clinical characteristics of the study population were analysed with descriptive statistics. In analysing the questionnaires, we used the following approach: when patients gave 2 answers on the same question or put a mark between 2 answers, the worse outcome was recorded. Questionnaires with missing items were excluded.

*Internal consistency reliability* of the two ArmA sections was assessed with Cronbach’s alpha. A Cronbach’s alpha > 0.80 was considered good, 0.80–0.70 was considered moderate, and < 0.70 was considered low [23].

*Test-retest reliability* was evaluated with the quadratic weighted kappa. Kappa ≥0.70 was considered to indicate good reproducibility [23, 24].

*Face and content validity* (relevance and adequacy of items for the intended use) was evaluated by letting a group of clinicians and experts in spasticity induced by CNS injury carefully review the prefinal version of ArmA-S. This version was also reviewed by 15 patients with UL spasticity due to SCI and stroke. After modifications, the final version of the ArmA-S was reviewed by 20 patients with spasticity induced CNS injury, who responded to a feasibility questionnaire that asked about time to complete the measure, ease of completion, relevance and usefulness of the questionnaire as a whole and the different sections (a passive, b active). Each question was rated on a five-point Likert scale. The final version was also sent to 8 clinicians who work with patients with disabling UL spasticity to ask about their perceptions of the relevance, comprehensiveness and usefulness of the ArmA-S. Clinicians answers were ratings on a five-point Likert scale or yes or no. Please see Additional file 2: a-b for more information on the feasibility questionnaire completed by patients and clinicians.

*The acceptability* of the questionnaire’s was assessed by missing data analyses and user feedback including the percentage of missing responses to survey questions, the distribution of scores, and the magnitude of ceiling and floor effects (i.e., proportion of best and worst possible scores, respectively). Floor and ceiling effects are considered to be present when more than 15% of the respondents reach the highest or lowest possible numeric value of a score. A high floor or ceiling effect could make it difficult to measure therapy-induced changes [23, 25].

To assess *Construct validity* a set of a priori hypotheses based on clinical experience were generated. We expected to find low to moderate correlation using Spearman’s rank correlation analyses (r_S_) between ArmA-S and three other clinical measures collected at baseline since the other measures were considered to cover somewhat different aspects as compared to ArmA-S. The correlation coefficients were interpreted according to an often-quoted rule of thumb: 0.90–1.00, very high; 0.70–0.90, high; 0.50–0.70, moderate; 0.30–0.50, low; and 0.00–0.30, little or none [26]. More specifically, we hypothesized that both of the ArmA-S subscales would have a low correlation with the DASH score; that subscale A would correlate moderately with the MAS composite score; and that subscale B would correlate moderately with the GRT. Structural and cross-cultural aspects of construct validity was not analysed in this study due to the small sample size.

To assess *responsiveness* a set of a priori hypotheses based on clinical experience were generated. First, we assessed the validity of therapy-induced change in outcome scores, referred to as longitudinal validity by hypothesizing that the change from pre- to postintervention in the total score of section A of ArmA-S would have a low correlation with the changes in DASH and GRT and a moderate correlation with the change in MAS. The pre- to postintervention change in the total score on section B of ArmA-S was further hypothesized to have a low correlation with the change in DASH and moderate correlation with the change in MAS and GRT. The change from baseline to the 3-month follow-up in outcome measures was calculated with the Wilcoxon signed-rank test.

*Interpretability* was judged from estimates of minimal important change (MIC). MIC was calculated the same two ways as in the psychometric analyses of ArmA [14], using a distribution-based method [27], and further, as half the baseline standard deviation for sections A and B as an estimation of MIC. This approach was applied to the whole study population, as well as to each of the three treatment regimens separately for both analyses. Both methods use parametric assumption and therefore provide only a preliminary indication of interpretability because ArmA-S is an ordinal measure. All data analyses were done with SPSS for MAC (Version 27: SPSS, Chicago, IL, USA).

## Results

### Study participants

The study sample consisted of 58 patients with debilitating UL spasticity due to SCI (*n* = 31), stroke (*n* = 25), TBI (*n* = 4), or other diagnosis (*n* = 6). Eight patients had undergone spasticity-correcting surgery on both the right and left arms, on different occasions, for a total of 66 interventions. The mean age of the patients was 57 years (range 19–79). The mean time since the injury was 8.1 years (range 1–26). Preoperative allocation to a treatment regimen was based on the residual volitional motor control in the UL and on cognitive ability: 25 patients were assigned to HFR (38%), 30 to LFR (45%), and 11 to NFR (17%). Demographic and clinical characteristics of the study participants are listed in Table 2. Of 66 collected questionnaires, five were excluded because planned surgeries were postponed, resulting in a maximum of 61 questionnaires for analyses. Fifty-one patients completed the questionnaire twice for test-retest reliability; however, three completed questionnaires were excluded for missing answers, leaving 48 questionnaires for the test-retest analyses. The average time range between survey 1 and 2 was 6.7 days (range 4–10 days).

**Table 2:** Demographic and clinical characteristics of the study population (*n* = 66)

Mean age (years) (min-max)	57 (19–79)
Male/ female ratio	44 (67)/22 (33)
Diagnosis	
Spinal cord injury	31 (47)
Stroke	25 (38)
Traumatic brain injury	4 (6)
Other	6 (9)
Affected arm (right/left)	37 (56)/29 (44)
Treatment regimen	
High-functioning regimen	25 (38)
Low-functioning regimen	30 (45)
Non-functioning regimen	11 (17)
Mean test/retest time interval (days) (min-max)	6.7 (4–10)
Time between injury and baseline years (min-max)	8.1 (1–26)

Data is reported as number (%) unless indicated otherwise

Min: minimum; Max: maximum; other diagnosis: multiple sclerosis, cerebral paralysis, spina bifida, Wilson disease

#### Translation and adaption

##### Initial forward translation of the ‘tryout’ version of ArmA

In our search for a questionnaire that is sensitive for change in a heterogenic population of patients with neurological injuries, the choice fell on ArmA. To make a preliminary feasibility assessment of ArmA in a Swedish clinical setting, the original English version of the questionnaire was first translated into Swedish by two bilingual clinicians using a forward translation procedure. This first version is referred to as the tryout-ArmA-S. Testing of the tryout-ArmA-S, which was originally developed for patients with unilateral hemiplegia, revealed that patients with bilateral UL motor impairment after SCI were confused by the term the *affected arm* as they had bilateral UL spasticity. Another confusion arose from the original instructions, which specified that the response option 0 (*no difficulty*) be selected if the activity is *never done*. However, this has nothing to do with the patient’s affected/treated arm, causing difficulties in selecting option 0 (*no difficulty*) versus option 4 (*unable to do*) and increasing the need for explanation in a face-to-face situation. Patients thought that most questionnaire items were meaningful. After using the tryout-ArmA-S for 18 months in our clinical setting, we decided to proceed with psychometric evaluation despite its shortcomings. We therefore conducted a proper back-translation procedure.

##### Back-translation procedure

The guide to completion and questionnaire items in ArmA were easily translated from English to Swedish. Item 10 in section B (*handle a home telephone*) was changed because such phones are rarely used in Sweden nowadays. It was replaced by the item *handle your phone*. Some additional minor adjustment was made in the demographic part of ArmA-S: SCI was added as a neurological condition, and information about the caregiver was expanded to include hours and type of assistance (caregiver or professional). To facilitate completion of the questionnaire by patients with bilateral UL motor impairment, the term *affected arm* in the ArmA-S was clarified by adding *the arm that will be, or is treated*. The most significant modification in the Swedish version of ArmA was done to minimize the risk of faulty/misleading responses when a specific activity was *never done* by patients. Misinterpretation could lead to false-negative results if the patient argues the activity was *never done* before surgery (which equals score 0, *no difficulty*) even though the true reason is the severely impaired UL and the postsurgery score is 1 (*no difficulty*) to 4 (*maximum difficulty*). Thus, although the patient improved after surgery, the scoring indicates the opposite. To help patients select proper responses, the option *never done* (score 0) was added to ArmA-S, resulting in a six-point Likert scoring system. Further, instead of presenting the response options as digits (0–4), we changed the Likert-scale to verbal statements, describing the degree of difficulty as ranging from no *difficulty* to *unable to perform*, which are converted to scores 0–4. Instead of circling a response digit, the respondent is asked to mark with an X the appropriate verbal statement for each activity.

### Validity of final ArmA-S

The *content validity* of final version of ArmA-S was judged to be good based on opinions from both patients and expert clinicians. Further, this version was recognized as having good *face validity* in the sense of being clear, understandable, and easy to complete. All patients except one (5%) responded that the final version was easy or moderately easy to complete, and all patients thought the questions were moderately to very relevant. With no exceptions, the clinicians responded that the measurement tool would be useful in clinical settings for patients with hemiplegia, but also for patient groups with other neurological injuries. Of the 20 patients who were timed, 10 (50%) completed the questionnaire in less than 10 min and 90% in less than 20 min. In the analyses of floor or ceiling effects, the baseline score before surgery was used. One (1.6%) of 61 completed questionnaires had the highest possible score (0 points) on section A, one (1.6%) had the highest score (0 points) on section B, and six (9.8%) had the worst possible score (52 points) on section B. Yet, the median (interquartile range) scores for sections A and B were 12 (8–17) and 46 (37–49), respectively. Thus, there were no floor or ceiling effects. The analyses of construct validity revealed great variety in the correlation between ArmA-S section A and B and the other outcome measures (Table 3). The GRT had the highest correlation with section B of the final ArmA-S (r_S_ = 0.59; *p* < 0.000), whereas DASH had little or no correlation with sections A and B (r_S_ = 0.05, *p* = 0.75 for both correlations).

**Table 3:** Association between ArmA-S Sections A and B and the outcome measures GRT, MAS and DASH at baseline, as well as for the change in scores from baseline to the three-month follow-up

Outcome measure	*n*	Baseline Score				Change in Score			
		Section A		Section B		Section A		Section B	
*Baseline*		r_S_	*p*	r_S_	*p*	r_S_	*p*	r_S_	*p*
MAS	48	.20	.216	.25	.120				
GRT	53	−.42	.003	.59	.000				
DASH	36	.05	.754	.05	.754				
*3-month follow-up*									
MAS change	40					.24	.138	−.20	.209
GRT change	48					.15	.302	−.45	.001
DASH change	35					.17	.323	.26	.128

*r*_*S*_ Spearman correlation coefficient, *ArmA-S* Arm activity measure Swedish version, *MAS* Modified Ashworth scale, *GRT* Grasp and release test, *DASH* Disabilities of the arm, shoulder, and hand

#### Reliability of the final ArmA-S

The *internal consistency* of the final ArmA-S version was high, with a Cronbach’s alpha coefficient of 0.94 for section A and 0.93 for section B (*n* = 61). Test-retest reliability, analysed for 48 patients, resulting in a quadratic weighted Cohen’s kappa coefficient of 0.86 (95% confidence interval [CI], 0.78–0.95) for section A and 0.83 (95% CI, 0.67–1.00) for section B. The *responsiveness* was analysed in patients who completed the survey before their spasticity-correcting surgery and 3 months afterward (*n* = 55). As hypothesized, assessment of *longitudinal validity* revealed little or low correlation between the mean change in the total score on section A and the mean change in all other outcome measures (DASH: r_S_ 0.2; *p* = 0.32; MAS: r_S_ 0.2; *p* = 0.14 and GRT: r_S_ 0.2; *p* = 0.30). The equivalent analysis for section B revealed little or low positive or negative correlation with the other measures (DASH: r_S_ 0.3; *p* = 0.13; MAS: r_S_ − 0.2; *p* = 0.21 and GRT: r_S_ − 0.2; *p* = 0.001). The analysis of the mean change in final ArmA-S total score from the pre-surgical survey to the 3-month follow-up (Table 4) showed significant increases in both section A and section B (*p* < 0.001). Eleven patients who reported little or no use of the hand before surgery (section B score, 49–52) had some active use of the hand, as captured by the lower section B score 3 months after surgery (30–48 in this subgroup). Significant improvements were also shown in the mean change in GRT and MAS (*p* < 0.001) but not DASH (*p* = 0.732).

**Table 4:** Changes in outcome measures between baseline and the 3-month follow-up

Outcome Measure	n	Baseline Median (IQR)	3 months Median (IQR)	Change Median (IQR)	*P*
**ArmA-S**					
*Section A Total score*	55	12.0 (8–17)	5.0 (2–9)	−6.0 (−1.0 – −10.0)	.000
HFR	17	12.0 (9.5–15.0)	5.0 (2.5–9.0)	−6.0 (−1.5 – −10)	.001
LFR	29	11 (6.0–14.0)	5.0 (1.0–-7.7)	−4.0 (−1.0 – −9.7)	.000
NFR	9	17.5 (12.7–18.5)	8.0 (3.75–--11.2)	−9.0 (−6.0 – −12.0)	.005
*Section B Total score*	55	46.0 (37–49)	42.0 (20–48)	−4.0 (−.0 – −13.0)	.000
HFR	17	37 (29.5–47.0)	28.0 (14.0–41.5)	−9.0 (−4.0 – −19.0)	.000
LFR	29	46.5 (39.2–48.7)	42.5 (25.7–47.7)	−4.0 (−0.2 – − 13.2)	.002
NFR	9	50.0 (39.0–52.0)	50.5 (37.5–52.0)	0.0 (0.0 – −1.2)	.343
GRT	48	14.5 (0.0–57.5)	23.0 (0.0–67.7)	.0 (.0–11.7)	.000
MAS	40	3.0 (2.7–4.0)	0.8 (0.2–1.2)	−2.2 (−1.8 – −3.0)	.000
DASH 1–21	35	8.0 (63–101)	83.0 (68–101)	.0 (−3.0–4.0)	.732

*IQR* Interquartile range, *ArmA-S* Arm activity measure Swedish version, *HFR* High-functioning regimen, *LFR* Low-functioning regimen, *NFR* Non-functioning regimen, *GRT* Grasp and release test, *MAS* Modified Ashworth scale, *DASH* Disabilities of the arm, shoulder, and hand

MIC was estimated with a distribution-based method and a criterion-based method (Table 5). For the study population as a whole using a distribution-based method the MIC for section A and B was shown to be 3.2 points and 6.8, respectively. Using a criterion-based method across the whole study population (*n* = 55) resulted in a decrease of 6.1 points in section A and a decrease of 6.5 in section B.

**Table 5:** Minimal important change estimated with a criterion-based approach for patients who underwent surgery or a distribution-based approach for patients who underwent surgery and had complete baseline measures

Method	*n*	ArmA-S Section A	*n*	ArmA-S Section B
Criterion-based approach				
Whole group	55	6.1	55	6.5
HFR	17	6.2	17	11.2
LFR	29	5.0	29	5.8
NFR	9	8.3/9.1	9	0.7
Distribution-based approach				
Whole group	61	3.2	61	6.8
HFR	21	2.6	20	6.2/6.5
LFR	29	3.3	28	5.2 (4.2^a^)
NFR	11	2.8	10	9.7 (1.8^a^)

^a^Score achieved when one outlier was removed

*ArmA-S* Arm Activity Measure Swedish version, *HFR* High-functioning regimen, *LFR* Low-functioning regimen, *NFR* Non-functioning regimen

When inspecting the data for analyses of responsiveness (pre- and postsurgical items of sections A and B of ArmA-S), we noted some highly questionable responses to questionnaire items, mainly in section B. Specifically, even though we had added the response option *never done* to the scoring system, quite a few patients selected the *never done* option before surgery (*no difficulty*), but had selected one of the response options *no, mild, moderate, severe difficulty*, or even *unable to do activity* after surgery. This indicates an unsuccessful outcome, which was not in accordance with the empirical experiences of patients’ capabilities after the surgical intervention. Thus, the content of the translated version of ArmA still seemed to entail uncertainty. In complementary explorative data analyses, we therefore applied a score transformation to data, based on known characteristics of patients.

##### Complementary explorative data analysis

In complementary analyses, we compared the original scores with the transformed scores. The scores were transformed as follows. If the pre-surgical score was 0 (*never done*) and the postsurgical score was 0 (*no problem*) or between 1 and 4 (various degrees of difficulty), the pre-surgical score was considered an error and was changed to score 4 (*unable to do*). This transformation required that functional status before surgery clearly indicate that the patient was unable to do the specific activity.

**Fig. 2:**
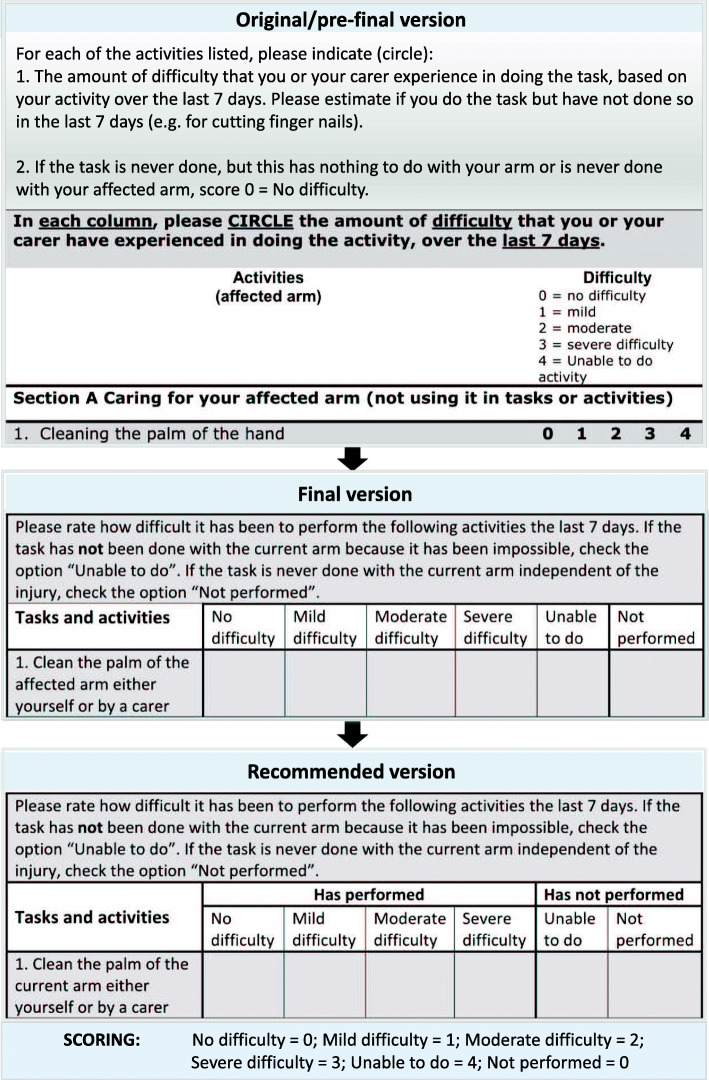
Descriptions of the modifications of the Likert-scale between the original/try-out version, final version, and the recommended version of ArmA-S

For all test-retest questionnaires, 40% of patients made this error. The results from the corrected analysis substantially lowered the CI for the adjusted scale, resulting in a quadratic weighted kappa coefficient of 0.91 (95% CI 0.85–0.97) for section A and 0.96 (95% CI 0.93–0.99) for section B. Therefore, in the recommended Swedish version, the guidance was clarified and the scale was altered to make it easier to complete the questionnaire correctly and independently and to minimize identified errors without changing the original instructions of the scale. See Additional file 3 for the recommended ArmA-S. (Fig. 2)

A complementary analysis was made in which the participants were split in two sub-cohorts based on diagnosis (SCI *n* = 18 and Stroke *n* = 20). Splitting the cohort resulting in a quadratic weighted Cohen’s kappa coefficient of 0.92 (95% confidence interval [CI], 0.85–0.99) for section A in the SCI group and 0.79 (95% CI, 0.62–0.97) in the stroke group. Corresponding figures for section B was 0.79 (95% CI, 0.51–1.07) and 0.82 (95% CI, 0.67–1.0), respectively. The analysis of the mean change in final ArmA-S total score from pre-intervention to the 3-month follow-up showed significant increases for both groups in both section A and B. When comparing the mean change in final ArmA-S total score between the subgroups a significant difference was demonstrated for section B (*p* = 0.016) in favour of the SCI -group, but not for section A (*p* = 0.116).

## Discussion

This study provides preliminary support for the use of ArmA-S to assess active and passive UL functional status in patients with disabling UL spasticity. The majority of the tested hypotheses were confirmed, demonstrating that the ArmA-S has good validity, reliability, and responsiveness in the evaluation of patients with UL spasticity due to neurological conditions, including SCI. Most psychometric properties were in agreement with the original English version of ArmA. In the previously evaluated English and Thai versions of ArmA, section A comprised 7 items [14–16]. Based on recommendation by the developer of ArmA, one additional item was added to section A [14]. The version used in the present study comprises 8 items in section A, (core range 0–32 points). Thus, there is a discrepancy in the maximum section A score between the current Swedish and the English and Thai versions of ArmA.

The translation and cultural adaptation of ArmA-S to ensure semantic and conceptual equivalence to the original version proceeded without difficulties. The questionnaire was judged by patients and clinicians to have *good content and face validity* as well as *acceptability*. Few missing answers were found indicating a good completeness of responses. Half of the patients completed ArmA-S in less than 10 min, which further supports its clinical feasibility and implementability. The somewhat longer completion time for some participants as compared to the versions in English [14] and Thai [16] may be due to differences in the underlying neurological injury among participants in the two studies.

We found that the ArmA-S had no floor or ceiling effects. Although the median score of the section B total score was rather high (47, IQR 37–49), only six patients (9.8%) had the highest possible score (maximum disability) versus 37% of respondents in the original ArmA. In the present study, 38% of the patients were treated with the HFR, suggesting that their active UL function was expected to improve after surgery. The lack of floor or ceiling effects in ArmA-S speaks in favour of the tool in this type of clinical setting. For the six patients who had the maximal score in section B (maximum disability), the spasticity-correcting surgery was aimed to facilitate passive caring activities, such as personal hygiene and dressing. The clinical outcome in the present study showed that patients with little or no active arm and hand function before surgery, as measured by ArmA-S, achieved gains from the intervention. The unique combination of active and passive activity aspects in ArmA makes the questionnaire useful for assessing improvements in activity and hygiene aspects in a heterogeneous group of patients with disabling UL spasticity.

In assessing the effectiveness of health care interventions, accurate determinations of the MIC is important. In a previous study of patients with chronic stroke [28], Lewek et al. suggested that expectations for changing gait speed be based on baseline gait speed. Thus, for patients with more significant gait impairment after stroke, one should not expect as large a change as for faster walkers. Lewek et al. also noted that although the change in gait speed is seemingly smaller for slower walkers, it still be a ‘real change’. Most importantly, Lewek and al. noted that a single MIC is often indiscriminately applied to all study participants to determine success, despite differences in participants’ potential treatment responses, as we also found. Like Lewek et al., we believe that treatment-induced gains in UL function may be more clinically relevant for patients with more severe functional impairment after neurological injuries. Consequently, different MICs should be applied to patient groups that vary in the degree of disability. However, our sample size is too small to determine MIC accurately and reliably. Moreover, the ordinal character of the ArmA-S hampers the stability definition of MIC across the scale, as well as the proper interpretation of the final results [29]. Therefore, we present only a preliminary indication of interpretability for ArmA-S.

We found that ArmA-S has high *internal consistency* in both section A and section B **(**0.94 and 0.93, respectively), in line with the previous psychometric investigations of original ArmA [16]. The analyses of *responsiveness* indicate that ArmA-S (in conformity with GRT and MAS) is better for detecting changes due to spasticity-correcting surgery as compared to DASH, which did not capture any significant improvement. This disparity is not surprising, as DASH was developed to assess a wide range of UL problems and not specifically spasticity-related disorders. Further, DASH was designed to assess higher-level function and is therefore likely to show significant floor effects in a neurologically impaired population. This was the case in a previous study investigating the effectiveness of botulinum toxin for patients with UL spasticity [14]. Section A of ArmA did reveal significant improvement after treatment with botulinum toxin, whereas DASH did not [14]. In DASH, the respondent is asked to report the degree of difficulty in performing various physical activities because of an arm, shoulder, or hand problem, but with no referral to a specific arm. This may lessen the sensitivity to change and has raised concerns about DASH as an outcome measure in patients with disability after stroke [29]. On the other hand, our findings support the use of ArmA-S in clinical or research contexts involving patients with UL spasticity. However, one must bear in mind that self-reporting of spasticity-related disability is a challenge, since patients often experience concurrent clonus, rigidity, and neuropathic pain along with the various aspects of spasticity. Discrimination among symptoms may be difficult [30–32]. Complementary evaluation of aspects such as body functions and grip ability is recommended to provide a more complete picture of complex disorders after neurological injuries.

As hypothesized, both ArmA-S section A and B correlated moderatly with GRT. Both sections showed low correlation with MAS and DASH. In contrast, the original ArmA did correlate with DASH [14]. This discrepancy may reflect differences in the clinical characteristics of the two study populations.

In the study evaluating the original version of ArmA [14], the majority of participants were stroke survivors. The complementary analyses in the present study in which the study cohort was split in two, demonstrate satisfactory test-retest reliability and responsiveness of the scale when used for individuals with SCI.

Our study had some limitations that must be considered. First, further investigation and larger sample sizes will be required to clarify the internal structure of the ArmA-S. Further, since this study did not assess cross-cultural validity of ArmA-S it is not recommended to compare scores on the original ArmA with those on the ArmA-S. Although the time span for the test-retest assessment was longer in this study (4–10 days) as compared to the previous evaluation of the original ArmA (1 day), it may still be considered a short enough time span to result in some recall bias. An accurate MIC is yet to be established. Moreover, it remains to be determined whether the MIC differs between groups of patients that vary in the degree of remaining neurological deficit. We did not record response rate in the present study. Most of the questionnaire were answered by patients in the clinic to increase the response rate as well as to make sure that all questions were answered. Having the questionnaire sent home to all patients would most likely have lowered the response rate and resulted in a higher degree of missing responses. Although patients and clinicians both found the questionnaire to be feasible and relevant, only clinicians were asked questions about comprehensiveness of the questionnaire. It would have been preferable to also ask the patients about the comprehensiveness of the measure. Further, some questions arose from both clinicians and patients that indicate areas for improvement of the ArmA-S as a stable self-report measure of spasticity-related UL disability.

## Conclusion

The ArmA-S is a valid and reliable measure for assessing passive and active function in patients with UL spasticity. It should help both researchers and clinicians monitor treatment-induced changes in UL function in patients with various degrees of neurological disability. Further validation in larger samples is needed to evaluate the measurement properties of ArmA-S in response to structural and cross-cultural validity, other treatments besides surgery and to determine the MIC for patients with various degrees of UL disability. Future studies should also focus on obtaining population norms for ArmA-S and on psychometric evaluation of the slightly modified and recommended version of ArmA-S.

## Supplementary Information

**Additional file 1.** Description of the study flow.

**Additional file 2.** Feasibility questionnaires sent out to participants and clinicians.

**Additional file 3.** Recommended version of ArmA-S.

## Data Availability

The dataset analysed during the current study are available from the corresponding author on reasonable request.

## Abbreviations

ADL: Activities of daily living
ArmA: Arm Activity Measure
ArmA-S: Arm Activity Measure Swedish version
DASH: Disability of the arm, shoulder and hand
CARE: Centre of Advanced Reconstruction of Extremities
CNS: Central nervous system
CI: Confidence interval
GRT: Grasp and release test
HFR: High functioning regimen
IQR: Interquartile range
LFR: Low functioning regimen
MAS: Modified Ashworth scale
MIC: Minimal important change
NFR: None functioning regimen
RS: Spearman’s rank correlation coefficient
SCI: Spinal cord injury
TBI: Traumatic brain injury
UL: Upper limb
