# Supplementary material for: Arm activity measure (ArmA): psychometric evaluation of the Swedish version
Source: J Patient Rep Outcomes. 2021 May 12;5:39. doi: 10.1186/s41687-021-00310-4 (PMC8116475; doi:10.1186/s41687-021-00310-4)
Supplement: Supplementary file 1 — Additional file 1. Description of the study flow. [file 41687_2021_310_MOESM1_ESM.docx]

**Appendix 1. Description of the study flow**

**STAGE 1. Project initiation**

In the project initiation phase, a literature search was done to find a useful self-report questionnaire for evaluation of both *active and passive aspects of daily activities* in patients with spasticity-related disability in the upper limb (UL). Requirements for the questionnaire were: (1) it could be completed by the patient at home, either independently or with assistance by a caregiver or professional; (2) it is suitable for patients with different diagnoses and levels of injury severity and; (3) it should be valid, reliable, and responsive to change. After a thorough literature search and gathering of expert opinions from clinicians at specialized spasticity centres around Sweden and rehabilitation centres for patients with tetraplegia worldwide, Arm Activity Measure (ArmA) was selected. A forward translation of ArmA from English to Swedish was done by two bilingual translators. The preliminary Swedish version was called ‘try-out ArmA-S’.

**STAGE 2. Feasibility assessment of try-out ArmA-S**

The try-out ArmA-S underwent a preliminary feasibility assessment in a Swedish clinical context among the target population for about 1.5 years. Patients identified difficulties in selecting appropriate responses in the scale. They had difficulty in separate the response option *unable to do activity* (equals score 4) from the response option *never done with my affected hand*, which according to the original instructions should be scored 0 (*no difficulty*). Patients with spinal cord injuries (SCIs) who had bilateral UL motor impairment were unsure about how to interpret the term *the affected arm* since both arms were affected.

**STAGE 3.** **Back-translation**

Approval for a proper translation was received from the developer of ArmA. The original questionnaire was translated from English to Swedish by two bilingual translators with medical background whose mother tongue was Swedish. The translations were done independently and named ArmA-translation 1 (ArmA-T1) and ArmA-translation 2 (ArmA-T2). The two version were reviewed and discussed with the aim to form a synthesis. Differences were resolved by consensus, and the new merged version was entitled ArmA-T12. The ArmA-T12 was translated back into English by two other bilingual translators whose first language was English (one with medical background and one without). They independently produced two English versions entitled ArmA-Back-translation 1 (ArmA-BT1) and ArmA-Back-translation 2 (ArmA-BT2).

**STAGE 4. Review by expert committee**

An expert panel was appointed to review ArmA-BT1 and ArmA-BT2. The panel included the two forward translators and two clinicians, one a native speaker of English. The backward translators were contacted for minor clarifications. The expert panel compared the two versions with the original English ArmA, considering semantic, idiomatic, experiential, and contextual aspects to identify errors and taking into account previous reasoning. Discrepancies were resolved by consensus, and an agreement was reached by panel members. The problems that were identified during stage 2 were discussed. The adaptations made to the questionnaire included the following: item 10 in section B (*handle a home telephone*) was changed to *handle your phone* since home telephones were judged to be rarely used in Sweden nowadays. Some additional minor adjustment was made in the demographic part of ArmA; SCI was added as a neurological condition, and the information about the caregiver was expanded to include hours and type of assistance (caregiver or professional). For the measure to be appropriate for patients with both uni- and bilateral UL disability, the wording was changed to *the current arm*, instead of *the affected arm*. The new version was entitled pre-final ArmA-S.

**STAGE 5. Feasibility assessment of pre-final ArmA-S**

*Face validity by clinicians and persons with lived experience*. Three clinicians working with patient with disabling UL spasticity were asked to comment about the suitability of ArmA-S in their clinical contexts, considering both the wording and intelligibility of the questionnaire. The pre-final version was administered to 9 patients with SCI and 6 with stroke to assess face validity and content validity. After filling out the questionnaire, patients were asked, in a face-to-face interview, for comments and suggestions about the content, clarity of wording, and relevance. The feedback from these patients was comparable to that received when field-testing the try-out version.

*Scaling issues.* By interviewing patients, we found that quite a few of them choose the response option 0 (*no difficulty*), since they were told to do so in the instructions if the activity was never done with current arm (especially for tasks usually done with the dominant hand, such as *write on paper*). When specifically asked for the reason to this, they responded that either *the task was impossible to accomplish after the injury*, or it was nowadays *easier to accomplish the task with the nondominant hand*. Patients also stated that for the item *cutting finger nails* they did not understand whether it meant cutting finger nails on the current UL or the contralateral. Since the comments were judged as highly relevant, they were taken in account as we further modified the questionnaire. The most noteworthy change was the addition of the response option *never done* (score 0) to ArmA-S, resulting in a six-point Likert scoring system. The modifications were judged to be crucial for use of ArmA-S as a self-report questionnaire, without the need of oral guidance. Another rationale for the changes was to achieve a measure that is sensitive to change after spasticity interventions, even though equivalence to the original version of ArmA was lost in part. The new version was called the ‘final version of ArmA-S’.

*Face validity of final ArmA-S*. After modifications, the final version of ArmA-S was reviewed by 20 patients with spasticity induced by CNS injury, who also responded to written questions about the questionnaire’s relevance and how time-consuming and easy it was to complete. The final version was also sent to 8 clinicians working with patient with disabling UL spasticity to enquire about their perception of relevance and usefulness of the ArmA-S

**STAGE 6. Psychometric evaluation**

The validity, reliability, responsiveness, and interpretability of the final version of ArmA-S were assessed as described in the Methods section.

**STAGE 7. Design of a recommended Swedish version of ArmA (ArmA-S)**

During the psychometric analyses, it was acknowledged that some additional adjustment was needed to further improve the feasibility (ease of completion) and most likely the validity of the measure. In case the respondent selected the response option *task is never done*, we added two options to specify the reason for not doing the task: (1) *because the individual is hindered to do the task after the injury* and (2) *the task has never been done with the current UL, either before or after the injury*. Thus, the layout of the Likert scale was somewhat modified to make it easier to distinguish between *unable to do* and *never don*e*.* The step-wise modification of the Likert scale and its verbal statements are presented in Figure 2.
