# Supplementary material for: Arm activity measure (ArmA): psychometric evaluation of the Swedish version
Source: J Patient Rep Outcomes. 2021 May 12;5:39. doi: 10.1186/s41687-021-00310-4 (PMC8116475; doi:10.1186/s41687-021-00310-4)
Supplement: Supplementary file 2 — Additional file 2. Feasibility questionnaires sent out to participants and clinicians. [file 41687_2021_310_MOESM2_ESM.zip › Appendix 3a_Feasability questionnaire patient.docx]

**Feasibility questionnaire (patients)**

We would appreciate if you could take the time to answer some questions regarding the questionnaire ArmA that you just filled in.

Circle the alternative you agree with

1. How long did it take to answer ArmA?

20 min or more 15-20min 10-15min 5-10min less than 5min

2.How easy was it to answer?

Very easy easy moderate difficult very difficult

3.How relevant did you think the questions were (both section a and b)?

Very relevant relevant moderate somewhat relevant irrelevant

4. How relevant did you think the questions in section A (passive functions) were?

Very relevant relevant moderate somewhat relevant irrelevant

5. How relevant did you think that the question in section B (active functions) were?

Very relevant relevant moderate somewhat relevant irrelevant

6. How clear did you think the instructions were?

Very clear clear moderate somewhat clear not clear

7. Other comments­­­­­­­­­­­________

___________________________________________________________________

­­­­­­­­­­­­­­­­­­­____________________________________________________________________

­­­­­­­­­­­­­­­­­­­­­­­­­____________________________________________________________________

­­­­____________________________________________________________________

­­­­­­­­­­­­­­­­­­­­­­­­­­­­­­­­­­­­­­­­­­­­­­­____________________________________________________________________

**Thanks**

­­­­­­­­­­­­­­­­­­­­­­­­­­­­­­­­­­­­­
