# Supplementary material for: Arm activity measure (ArmA): psychometric evaluation of the Swedish version
Source: J Patient Rep Outcomes. 2021 May 12;5:39. doi: 10.1186/s41687-021-00310-4 (PMC8116475; doi:10.1186/s41687-021-00310-4)
Supplement: Supplementary file 2 — Additional file 2. Feasibility questionnaires sent out to participants and clinicians. [file 41687_2021_310_MOESM2_ESM.zip › Appendix 3b_Feasibility questionnaire clinicians.docx]

**Feasibility questionnaire (clinicians)**

For how long have you been working with patients with spasticity problems?

What is your profession? _____________________

At which clinic/hospital do you work? ______________________________

Estimate the percentage of the following neurological diagnosis at your clinic?

Stroke: ____% Spinal cord injuries:___% Traumatic brain injuries:___% Other:___%

1. How clear do you consider the instructions in ArmA are?

Very clear Clear Moderate Unclear Very unclear

2. How clear do you think the scale in ArmA are?

Very clear Clear Moderate Unclear Very unclear

3. How relevant do you consider the questions in ArmA (section A and B) are? (target group; focal spasticity interventions in the arm)

Very relevant Relevant Moderate Irrelevant Very irrelevant

Please comment your answer_________________________________________

4. How relevant do you consider the questions in section a (passive functions) are?

Very relevant Relevant Moderate Irrelevant Very irrelevant

5. How relevant do you consider the questions in section b are (active functions)?

Very relevant Relevant Moderate Irrelevant Very irrelevant

6. In a Swedish context, did you miss any questions?

Yes No

If yes, which? _________________________________________________

___________________________________________________________________

7. In a Swedish context, do you consider any questions irrelevant?

Yes No

If yes, which? __________________________________________________________

___________________________________________________________________________

8. Do you believe ArmA would be useful in your clinic?

Yes No

9. ArmA is mainly developed for patients with spastic hemiplegia after stroke. Do you think it would be useful for patients with spasticity due to other neurological diagnosis?

Yes No

Please comment your answer ______________________________________________________________________________________________________________________________________

9. Other comments: ___________________________________________________

_______________________________________________________________________

**Thanks**
