# Supplementary material for: Arm activity measure (ArmA): psychometric evaluation of the Swedish version
Source: J Patient Rep Outcomes. 2021 May 12;5:39. doi: 10.1186/s41687-021-00310-4 (PMC8116475; doi:10.1186/s41687-021-00310-4)
Supplement: Supplementary file 3 — Additional file 3. Recommended version of ArmA-S. [file 41687_2021_310_MOESM3_ESM.pdf]

# A r m A - S

## Arm Activity Measure- S

Svensk version: T. Ramström, L. Bunketorp-Käll, J. Wangdell, 2020. C.A.R.E., Sahlgrenska Universitetssjukhuset/Mölndal, Mölndal.
Engelsk version: S. Ashford, King’s College London & Regional Rehabilitation Unit, Northwick Park Hospital, London.

# ArmA-S Arm Activity Measure, Svensk version

**Bakgrundsdata**

**Efternamn:** ______________________________ **Förnamn:** _________________________________

**Datum:** ______________________________

**Vänligen markera vem som fyller i frågeformuläret:**

- ☐ Självständigt
- ☐ En vårdare (familjemedlem)
- ☐ En vårdare (inte familjemedlem)
- ☐ Du och din vårdare (familjemedlem) tillsammans
- ☐ Du och din vårdare (inte familjemedlem) tillsammans
- ☐ Du med hjälp av en annan person (inte vårdare)

**Denna del av frågeformuläret berör allmän information om dig och dina (eventuella) vårdare.**

| **Ålder (år)**: | **Vilken hjälp har du i dag?** |
|---|---|
| **Kön:** **Man** ☐ **Kvinna** ☐ | ☐ Ingen ☐ Hemtjänst<br>☐ Personlig assistans ☐ Anhöriga<br><br>Om du har hjälp, ange hur många timmar per vecka?<br><br>………….. timmar (cirka) |

**Min neurologiska skada beror på:**

☐ Stroke ☐ Traumatisk hjärnskada ☐ Ryggmärgsskada
☐ Anoxisk hjärnskada ☐ Tumör ☐ Annat

**Om annat, vänligen uppge vad: ………………………………**

**Vilken arm är påverkad?** ☐ Höger ☐ Vänster ☐ Båda

**Innan skadan, var du:** ☐ Högerhänt ☐ Vänsterhänt ☐ Båda

# Instruktioner för ifyllnad av ArmA-S

**Sektion A frågar om *"omvårdnad" av din påverkade hand* (den hand som skall behandlas), antingen självständigt med den andra armen, med hjälp av någon annan, eller en kombination av de båda. Denna del berör inte hur du använder din påverkade arm för att genomföra någon av uppgifterna.**

**Sektion B frågar efter *vad du kan göra med din påverkade arm*, eller med båda armarna.**

**Svara på var och en av frågorna genom att kryssa i:**

**A.** Hur svårt du eller den som hjälper dig har att genomföra aktiviteten, baserat på en sammanfattning av de senaste 7 dagarna. Om det är någon aktivitet du brukar utföra, men inte utfört de senaste 7 dagarna (exempelvis att klippa naglarna) så får du uppskatta svårigheten med hur det brukar vara.

**B.** Om aktiviteten aldrig utförts av din påverkade arm, uppskatta hur det vore att utföra aktiviteten enligt instruktioner nedan:

- Om du aldrig har utfört aktiviteten *efter skadan*, men uppskattar att det vore omöjligt att utföra denna så markera med ett kryss i rutan "Omöjligt att utföra".

- Om du aldrig har utfört aktiviteten med aktuell arm, *varken före eller efter skadan* så markera med ett kryss i rutan "Aldrig utfört" (med aktuell arm).

**Om du inte kan fylla i frågeformuläret självständigt kan du:**

- Få hjälp av vårdare eller sjukvårdspersonal att fylla i (men du dikterar svaren).
- Få hjälp från en vårdare eller sjukvårdspersonal för att underlätta förståelse och svara på en fråga i taget.
- Få hjälp av en vårdare som kan fylla i frågeformuläret på dina vägnar baserat på svårigheterna denne upplever att aktiviteterna innebär.

Svensk version: T. Ramström, L. Bunketorp-Käll, J. Wangdell, 2020. C.A.R.E., Sahlgrenska Universitetssjukhuset/Mölndal, Mölndal.
Engelsk version: S. Ashford, King's College London & Regional Rehabilitation Unit, Northwick Park Hospital, London.

# ArmA-S svenskt protokoll sektion A

Efternamn: ___________________________ Förnamn: ______________________________

**ArmA-S sektion A.** Denna sektion handlar om **omvårdnaden** av din påverkade arm/hand =den hand som skall/ har behandlats, inte om användningen av handen i aktiviteter.

Gradera **hur svårt det varit** att utföra följande aktiviteter under de senaste 7 dagarna genom att kryssa för ett svarsalternativ för varje fråga. Om aktiviteten **aldrig utförts med aktuell arm efter skadan - skatta ”omöjligt att utföra”.** Om aktiviteten **aldrig utförts med aktuell arm före eller efter skadan - skatta ”aldrig utfört”.**

| | **Utför** | | | | **Utför aldrig** | |
|---|---|---|---|---|---|---|
| **Aktiviteter** | Ingen svårighet | Viss svårighet | Måttlig svårighet | Stor svårighet | Omöjligt att utföra | Aldrig utfört |
| 1. Tvätta den påverkade armens handflata, själv eller med hjälp av en annan person | | | | | | |
| 2. Klippa naglarna på den påverkade handen, själv eller med hjälp av en annan person | | | | | | |
| 3. Tvätta armhålan på den påverkade armen, själv eller med hjälp av en annan person | | | | | | |
| 4. Tvätta armbågsvecket på den påverkade armen, själv eller med hjälp av en annan person | | | | | | |
| 5. Lägga den påverkade armen på en kudde som ett stöd i sittande, själv eller med hjälp av en annan person. | | | | | | |
| 6. Trä den påverkade armen genom en tröj- eller skjortärm, själv eller med hjälp av en annan person | | | | | | |
| 7. Ta på en fingerhandske på den påverkade handen, själv eller med hjälp av en annan person | | | | | | |
| 8. Ta på en ortos på den påverkande armen, själv eller med hjälp av en annan person | | | | | | |

**Totalpoäng sektion A:** __________poäng. (Maximal poängsumma: 32 poäng)

**Skala:** Ingen svårighet=0, Viss svårighet=1, Måttlig svårighet= 2, Stor svårighet= 3, Omöjligt att utföra= 4, Aldrig utfört= 0

Svensk version: T. Ramström, L. Bunketorp-Käll, J. Wangdell, 2020. C.A.R.E., Sahlgrenska Universitetssjukhuset/Mölndal, Mölndal.
Engelsk version: S. Ashford, King’s College London & Regional Rehabilitation Unit, Northwick Park Hospital, London.

# ArmA-S svenskt protokoll sektion B

**Efternamn:** ______________________________ Förnamn: ________________________________

**ArmA-S sektion B** handlar om vad du **självständigt** kan utföra för aktiviteter genom att använda din påverkade arm/hand =den hand som skall/ har behandlats. Opåverkade hand/arm= den hand som ej har/ska behandlas.

Gradera din **förmåga** att utföra aktiviteter under de senaste 7 dagarna genom att kryssa för ett svarsalternativ för varje fråga. Om aktiviteten **aldrig utförts med aktuell arm efter skadan - skatta ”omöjligt att utföra”.** Om aktiviteten **aldrig utförts med aktuell arm före eller efter skadan - skatta ”aldrig utfört”.**

| | **Utför** | | | | **Utför aldrig** | |
|---|---|---|---|---|---|---|
| **Aktiviteter** | Ingen svårighet | Viss svårighet | Måttlig svårighet | Stor svårighet | Omöjligt att utföra | Aldrig utfört |
| 1. Svårighet med balansen när du går på grund av din arm | | | | | | |
| 2. Hålla ett objekt stilla medan du använder din opåverkade hand | | | | | | |
| 3. Öppna en sedan tidigare öppnad burk, med din påverkade arm | | | | | | |
| 4. Lyfta ett glas, flaska eller burk, med din påverkade arm | | | | | | |
| 5. Dricka ur en kopp eller mugg, med din påverkade arm | | | | | | |
| 6. Borsta tänderna, med din påverkade arm | | | | | | |
| 7. Stoppa in din tröja/skjorta, med din påverkade arm | | | | | | |
| 8. Skriva med penna, med din påverkade arm | | | | | | |
| 9. Äta med kniv och gaffel | | | | | | |
| 10. Slå ett telefonnummer på din telefon med din påverkade arm | | | | | | |
| 11. Knäppa upp knappar på kläder | | | | | | |
| 12. Kamma eller borsta håret, med din påverkade arm | | | | | | |
| 13. Med nyckel låsa upp en dörr, med din påverkade arm | | | | | | |

**Totalpoäng sektion B:** __________poäng. (Maximal poängsumma: 52 poäng)

**Skala:** Ingen svårighet=0, Viss svårighet=1, Måttlig svårighet= 2, Stor svårighet= 3, Omöjligt att utföra= 4, Aldrig utfört= 0

Svensk version: T. Ramström, L. Bunketorp-Käll, J. Wangdell, 2020. C.A.R.E., Sahlgrenska Universitetssjukhuset/Mölndal, Mölndal.
Engelsk version: S. Ashford, King’s College London & Regional Rehabilitation Unit, Northwick Park Hospital, London.
